# Cancer research, treatment, and COVID-19

**DOI:** 10.1186/s12645-020-00063-7

**Published:** 2020-08-06

**Authors:** Steven A. Curley, Frederick Currell, Sunil Krishnan, Zeljka Krpetic

**Affiliations:** 1 CHRISTUS Mother Frances Hospital – Tyler, 800 East Dawson Street, Tyler, TX 75701 USA; 2 The Dalton Cumbrian Facility and the School of Chemistry, The University of Manchester, Manchester, M13 9PL UK; 3 Department of Radiation Oncology, Mayo Clinic Florida, Jacksonville, FL USA; 4 Biomedical Research Centre, School of Science, Engineering and Environment, University of Salford, Manchester, M5 4WT UK

The COVID-19 pandemic has radically changed the world we live in. The novel coronavirus SARS-CoV-2, the infectious agent that causes the disease, is believed to have first appeared around December 2019, and in the months since then it has wreaked havoc on human communities, health systems, and economies worldwide. At the time of writing, it is estimated that more than 12 million individuals have been diagnosed with COVID-19, while approximately 550,000 deaths are attributed to the disease (COVID-19 Dashboard 2020). This has been a topic of constant and intense conversation, quite appropriately.

Equally so, there has been a shift in priorities from cancer prevention, treatment and research to COVID-19 prevention, treatment and research. As with rebalancing our investment portfolios during a financial crisis, we view the current landscape of shifting priorities as an opportunity to assess and equilibrate our twin interests in securing the quality and quantity of life of our patients and advancing new science with that of navigating the COVID-19 crisis responsibly and resolutely.

Extrapolating from data available from 2018 (Cancer fact sheet 2018), approximately 9 million patients have been newly diagnosed with a malignant disease in the first half of 2020. During that same timeframe, there have been no fewer than 4.75 million deaths from cancer throughout the world. At least 43 million people are currently living with a diagnosis of cancer and are undergoing a variety of treatments. Clearly, the impact from cancer far outstrips that of COVID-19, but does not garner the attention nor sense of panic among governments, businesses, the media, and communities. We do not dispute the importance of taking necessary and sometimes radical actions to prevent the spread of an infectious disease such as COVID-19, but we must remember as individuals involved in looking for better ways to prevent, diagnose, and treat malignant diseases that the current impact of cancer on health worldwide far surpasses that of COVID-19.

Cancer is the second leading cause of death globally and its incidence is increasing. As the size of the human population expands worldwide, the number of new cases and deaths from cancer annually continues to increase. Cancer can be caused by genetic or environmental factors, but also by infectious diseases, such as hepatitis B or C virus or human papilloma virus. Furthermore, the COVID-19 crisis has directly impacted the care of cancer patients. One of the co-authors of this editorial (SK) has recently submitted a paper looking at the effect of COVID-19 on cancer care worldwide (Venkatesulu et al. 2020). In this meta-analysis, patients who are being treated for cancer and who develop COVID-19 have a higher likelihood of intubation and mortality. The highest mortality risk was among patients with hematologic malignancies or lung cancer, but we must remember that many patients being treated for cancer are immunosuppressed to some degree owing to their disease-related poor nutritional and performance status, cytotoxic chemotherapy, radiation treatments, or major surgical procedures.

As editors of *Cancer Nanotechnology*, we are committed to supporting and publishing cutting-edge research using nanotechnology as a tool to diagnose and treat patients with malignant disease. As an interdisciplinary community at the interface of biological science and nanotechnology, we must also use our resources and knowledge as scientists to collaborate with our colleagues who are involved in the extremely important work of developing enhanced COVID-19 diagnostics, therapeutics, and possibly a vaccine. The HIV crisis of the 1980s and 1990s demonstrated the ability of scientists working around the world to develop an understanding of the role of a retrovirus in causing AIDS. This collaboration led to the development of successful antiviral therapies, with the result that, while there is still no vaccine for HIV, infection with the virus is no longer a death sentence.

Through similar interdisciplinary collaborations, we remain hopeful and optimistic that we can support and assist our colleagues trying to develop a vaccine active against SARS-CoV-2—indeed, some of the underlying concepts and techniques associated with cancer treatment and nanotechnology can be readily expanded to support this endeavor. As a virus can be nanotechnology and considered a naturally occurring nanoparticle, falling into the nanometer size range, we are in the advantageous position of being able to translate our knowledge and contribute to the scientific development around COVID-19 treatment, prevention and diagnosis and, in particular, to advise on safety. Conversely, research put to use in the current pandemic has potential to carry across to cancer research. For example, nanotechnology has a tremendous potential to mitigate some issues caused by COVID-19, in particular in development of advanced personal protective equipment (PPE), allowing vulnerable patients to continue receiving treatment for their cancer while the pandemic is ongoing.

It is clear that it is an extraordinary time for scientists around the world, some who are working at the forefront of trying to find a treatment for COVID-19 or vaccine for the virus causing it, some whose research has been repurposed to focus on this field, and some who have simply had to put their research on hold because of lockdowns and laboratory closures. We recognize the hardships that a lot of our authors, reviewers and editorial board members face at this time, and we will therefore take a few small steps that we hope will help in some way:

- We will be flexible on deadlines for reviews. Reviewers requiring additional time should contact the editorial office or managing editor;
- Where revisions have been requested that require additional, non-essential experiments to be performed, but where the authors cannot currently access their laboratories, we are prepared, with guidance from our reviewers, to re-evaluate whether the requested experiments are necessary for publication;
- Where revisions have been requested that require essential experiments to be performed, our teams will work with a more relaxed timeline for completion of the revisions, given the extenuating circumstances that many laboratories face;
- If research that relates to COVID-19 is submitted to the journal, and is within scope, we will endeavor to have the research reviewed rapidly, while maintaining quality standards, to ensure the research to be disseminated quickly to the community. To this end, we remind authors that *Cancer Nanotechnology* encourages posting of preprints of primary research manuscripts on preprint servers. Authors submitting to the journal alternatively have the opportunity to make use of Springer Nature’s free *In Review* service on submission, which allows direct depositing of the research on our partner platform Research Square.

There is no doubt that the COVID-19 pandemic will have long-term effects on the landscape of research, potentially with greater focus on virology and preventing the next pandemic. However, we feel it is important that the scientific and medical community remain mindful that cancer is a substantial cause of disease and death throughout the world. The community around *Cancer Nanotechnology* must therefore continue to seek increased funding opportunities to promote cancer research and push for better cancer prevention, diagnostic, and treatment systems to make them more readily available and affordable for all people throughout all socioeconomic classes in the world. While continuing to perform our public health duties and working for the greater good, we must work together to resolutely maintain focus on championing evidence-based, patient-facing cancer prevention, diagnosis and treatment approaches.

COVID-19 is a disease that has undoubtedly wrought havoc on people’s lives—likewise cancer. We owe it to patients not to forget about them, to make their lives more bearable, and to give them a chance to live another day.

## Data Availability

Not applicable.
